# Household Hints and Recipes

**Published:** 1881-04

**Authors:** 


### Household Hints & Recipes

THAT POTATO SALAD.—The editor of this journal has obtained some little local notoriety for making a potato salad. Indeed, he carried away the prize in a recent potato-salad tournament, and this is the mixture that "did the business."

Of course, the possibility of making a good salad of any sort depends upon a properly constructed sauce. So we will begin with making what our lady friends call a "Mayonnaise Sauce." The entire secret of making this somewhat difficult emulsion is in having all the materials *cold* and adding the oil slowly. To attain the first, put the eggs and oil in a cool place for several hours before you need them. The second is managed by tightly corking the oil bottle, having first cut a little groove in the cork, thus allowing only a small stream of oil to escape at a time. Ingredients: The yolk of one raw egg, one pint of olive oil, vinegar or lemon juice, pepper and salt to taste. Put the egg in a soup plate (in very hot weather use a hot water plate, substituting ice water for the hot water), add a saltspoon of salt, stir a little with a silver fork; then commence adding drop by drop the oil, stirring the while; continue this till all the oil is used. After the sauce is once fairly started, the oil can be added rather more rapidly; but great care must be taken always to stir the same way, and thoroughly incorporate each installment of oil before more is added. If it should curdle, begin again with another yolk of egg and another plate. As soon as the new sauce is well started, stop adding oil, and add the curdled sauce by degrees till it is all used. Now, add good, sour vinegar until the emulsion becomes of the consistency of heavy cream. For chicken salad, use it plain; but for lobster, shrimp or crab, add a little French mustard.

Cut your cold, boiled potatoes into irregular chunks, the size of the end of your thumb, and in quantity to fill a quart measure. Of celery and cold boiled eggs, similarly prepared, one pint each. Mix them well in your salad bowl. Sprinkle with salt and red pepper, also with a teaspoonful of grated onion. (If you are fond of it, add more onion.) Now pour the sauce over the mixture and thoroughly incorporate the ingredients by carefully stirring—there, you have it.

FURNITURE POLISH.—Linseed oil, 2 pints; alcohol, ½ pint; vinegar ½ pint; butter of antimony, 2 ounces; spirits of turpentine, ½ pint. Shake well before using, and apply with a woolen rubber.

---

TO FIX PENCIL MARKS.—To fix pencil marks so they will not rub out, take well skimmed milk and dilute with an equal bulk of water. Wash the pencil marks (whether writing or drawing) with this liquid, using a soft, flat camel hair brush, and avoiding all rubbing. Place upon a flat board to dry.

---

RUST IS readily removed from white goods by soaking the stains in a weak solution of tin chloride, and rinsing immediately with much water. The tin salt is more reliable in removing iron rust, and quicker in its action than oxalic acid, unless the stains are soaked in a solution of the latter contained in a tin spoon, when the stains disappear in a shorter time.—*Pharm. Centralb.*

---

A GOOD ELASTIC GLUE.—Dissolve two ounces of India rubber in half a gallon of mineral naphtha. When the solution has been effected, add four ounces of shellac to the naphtha, place it in an iron vessel, apply heat cautiously, stir until well mixed, and then pour it upon a slab to cool. This can be melted at the same heat as common glue, can be applied with a brush, sets quickly, is elastic, and perfectly soluble in water.

---

CEMENT FOR LEATHER.—One who has tried everything, says that after an experience of fifteen years he has found nothing to equal the following as a cement for leather belting: Common glue and isinglass, equal parts, soaked for ten hours in just enough water to cover them. Bring gradually to a boiling heat and add pure tannin until the whole becomes ropy or appears like the white of eggs. Buff off the surfaces to be joined, apply this cement and clamp firmly.

---

TRANSPARENT PAINT FOR GLASS.—The following is taken from our back numbers: Take for *blue* pigment, Prussian blue; for *red*, crimson lake; for *yellow*, Indian yellow; and for other shades, a mixture of the appropriate primary colors. Rub them in a size made as follows: Venice turpentine, 2 parts; spirit of turpentine, 1 part; and apply with a brush. The colors are moderately fast unless exposed too long to direct sunlight. A solution of the various aniline dyes in shellac varnish has also been recommended.

---

DISTINGUISHING THE DIAMOND.—M. Robinet, of the French Academy, gives the following test for distinguishing colorless gems from diamonds. If a person looks through a transparent stone at any small object, such as a point of a needle, or a little hole in a card, and sees two small points, or two small holes, the stone is not a diamond. All white colorless gems, with the exception of the diamond, make the object examined appear double; in other words, double refraction, whenever exhibited by a stone, is proof conclusive that it is not a diamond.

---

DIAMOND INK.—The following remarks by Mr. F. L. Slocum will complement the note on the above subject in December, 1880, page 222:

Liquid hydrofluoric acid etches glass, leaving a smooth surface; the fumes of the acid, however, act on glass, leaving a slightly rough surface.

Ammonium fluoride dissolved in water etches a still rougher surface on slight heating; but if this salt be mixed with an equal bulk of barium sulphate, moistened with water and gently heated, a very rough and opaque surface is produced.

---

DRILLING GLASS.—The following directions were contributed to *Design and Work* by an optician: First make a saturated solution of camphor in spirits of turpentine; then make a *pear-shaped drill* the size of hole required; heat the drill to a white heat and plunge into mercury, and it will then be *very hard;* sharpen on an oilstone, knock drill in a brad-awl, dip the end of drill into the above solution, and work it as if you were working it through wood. It is no use fixing the drill in a drill-stock, because the motion all one way won't do. Keep the drill well moistened with the solution, and sharpen it when blunt. A file dipped into the solution will file the hole larger, and will not get blunt.

---

TALLOW FOR THE CARPET-BEETLE.—A writer in the *Germantown Telegraph* says: "It has been ascertained that the bacon-beetle (*Dermestes lardarius*), an insect closely allied to the carpet-beetle, will not touch its food when tallow is placed near it. A lady once placed in a jar a number of the larvæ of this beetle, together with the meat on which they were found feeding. When all of them were busied in devouring the meat, a small piece of tallow was placed near it, when the larvæ quickly left their food and huddled together in one side of the jar; nor would they come near the meat so long as the tallow remained near it. And when greatly pressed with hunger they began to devour each other, until only one was left, and this one died of starvation rather than to go near the tallow in order to get at its food.

"If it is found that the carpet-beetle has the same repugnance to tallow as the bacon-beetle has—and I have no doubt that it has—then we can easily protect our carpets from the attacks of this little pest by a judicious use of tallow. This latter substance is known to protect clothes from the attacks of the clothes-moth, and being easily obtained is far preferable to camphor, tobacco, etc. Before the carpets are laid down the floor should be painted over with melted tallow, taking care to fill up all the crevices in the floor, as it is in these that the larvæ usually conceal themselves."

---

THE PRESERVATION OF THE TEETH.—The following are the directions regarding the care of the teeth, issued by the Medical Committee of the National Dental Hospital, London:

1. The teeth should be cleaned at least once a day, the best time being night—last thing. For this purpose use a soft brush, on which take a little soap, and then some prepared chalk, brushing up and down and across. There is rarely any objection to the friction causing the gum to bleed slightly.

2. Avoid all rough usage of the teeth, such as cracking nuts, biting thread, etc., as by so doing even good, sound teeth may be injured.

3. When decay is first observed, advice should at once be sought. It is the stopping in a small hole that is of the greatest service, though not unfrequently a large filling preserves the tooth for years.

4. It is of the greatest importance that children from four years and upwards should have their teeth frequently examined by the dental surgeons, to see that the first set, particularly the back teeth, are not decaying too early; and to have the opportunity of timely treatment for the regulation and preservation of the second set.

5. Children should be taught to *rinse* the mouth night and morning, and to begin the use of the tooth-brush early (likewise the tooth-pick.)

6. With regard to the food of children, to those who are old enough, whole meal bread, porridge and milk should be given. This is much more wholesome and substantial food than white bread.

7. If the foregoing instructions were carried out, comparatively few teeth would have to be extracted.

